# A case of a woman with late-pregnancy-onset DKA who had normal glucose tolerance in the first trimester

**DOI:** 10.1530/EDM-13-0085

**Published:** 2014-04-01

**Authors:** Hiromi Himuro, Takashi Sugiyama, Hidekazu Nishigori, Masatoshi Saito, Satoru Nagase, Junichi Sugawara, Nobuo Yaegashi

**Affiliations:** Department of Obstetrics and GynecologyTohoku University Graduate School of Medicine1-1 Seiryo-machi, Aoba-ku, Sendai, Miyagi, 980-8574Japan

## Abstract

Diabetic ketoacidosis (DKA) during pregnancy is a serious complication in both mother and fetus. Most incidences occur during late pregnancy in women with type 1 diabetes mellitus. We report the rare case of a woman with type 1 diabetes mellitus who had normal glucose tolerance during the first trimester but developed DKA during late pregnancy. Although she had initially tested positive for screening of gestational diabetes mellitus during the first trimester, subsequent diagnostic 75-g oral glucose tolerance tests showed normal glucose tolerance. She developed DKA with severe general fatigue in late pregnancy. The patient's general condition improved after treatment for ketoacidosis, and she vaginally delivered a healthy infant at term. The presence of DKA caused by the onset of diabetes should be considered, even if the patient shows normal glucose tolerance during the first trimester.

**Learning points:**

- The presence of DKA caused by the onset of diabetes should be considered, even if the patient shows normal glucose tolerance during the first trimester.
- Symptoms including severe general fatigue, nausea, and weight loss are important signs to suspect DKA. Findings such as Kussmaul breathing with ketotic odor are also typical.
- Urinary test, atrial gas analysis, and anion gap are important. If pH shows normal value, calculation of anion gap is important. If the value of anion gap is more than 12, a practitioner should consider the presence of metabolic acidosis.

## Background

Diabetic ketoacidosis (DKA) is an acute metabolic complication (1). During pregnancy, it is associated with maternal and fetal mortality and requires immediate medical attention. Approximately 1–2% of pregnant women with impaired glucose tolerance experience DKA (2). Pregnancy-related DKA mainly occurs in women with type 1 diabetes mellitus and to a lesser extent in women with type 2 diabetes mellitus, gestational diabetes, and even newly diagnosed type 1 diabetes mellitus. With improved clinical care, including administration of insulin analogs and continuous s.c. insulin infusion both antepartum and intrapartum, the incidence of pregnancy-related DKA has gradually decreased. However, it remains a critical problem because it tends to occur at blood glucose levels that are lower than those in non-pregnant diabetic women (3). Furthermore, case reports of fulminant type 1 diabetes mellitus have been recently reported (4). We report the rare case of a woman with type 1 diabetes mellitus who had normal glucose tolerance during the first trimester but developed DKA during late pregnancy.

## Case presentation

A 32-year-old Japanese woman (3G2P) in her 28th week of gestation presented with a 3-week history of general fatigue and increased thirst. She had not been diagnosed with abnormal glucose tolerance before pregnancy, and she had no family history of diabetes mellitus. At the age of 20 years, she underwent unilateral tubectomy because of ectopic pregnancy.

After she became pregnant naturally, she received prenatal care in a private clinic. Her random plasma glucose level was 140 mg/dl at 8 weeks of gestation, but a 75-g oral glucose tolerance test (75-g OGTT) at 11 weeks showed that her glucose tolerance was normal. At 28 weeks of gestation, she was referred to our hospital because of severe general fatigue and a random plasma glucose level of 489 mg/dl. Her pre-pregnancy BMI was 19.9. Her blood pressure was 110/84 mmHg, heart rate 106 beats/min, and body temperature 36.5 °C. Physical examination showed Kussmaul breathing with ketotic odor. The laboratory findings are summarized in Table 1. Importantly, anion gap showed 21.9, suggesting presence of metabolic acidosis. No obvious non-reassuring fetal status was recognized.

**Table 1:** Laboratory findings at admission

	
CBC	
White blood cells	7300/μl
Red blood cells	417×10^4^/μl
Hemoglobin	12.4 g/dl
Hematocrit	36.0%
Platelet	20.4×10^4^/μl
Carbohydrate metabolism	
Glucose	348 mg/dl
Glycoalbumin	45%
HbA1c	13.6%
Anti-GAD antibody	25.0 U/ml
Anti-IA-2	1.5 U/ml
CPR	1.3 μg/l
1.5AG	1.3 μg/l
Biochemical test	
TP	6.6 g/dl
Albumin	3.4 g/dl
T-bilirubin	0.5 mg/dl
AST	13 IU/l
ALT	11 IU/l
LDH	170 IU/l
ALP	245 IU/l
Amylase	72 IU/l
CK	43 IU/l
BUN	10 mg/dl
Creatinine	0.49 mg/dl
UA	6.5 mg/dl
Na	132 mEq/l
K	3.8 mEq/l
Cl	98 mEq/l
Ca	8.8 mg/dl
CRP	0.1 mg/dl
Atrial blood gas	
pH	7.450
pCO_2_	17.1 mmHg
pO_2_	120 mmHg
HCO_3_	12.1 mmol/l
BE	−9.8 mmol/l
Urinary test	
pH	5.5
Protein	1+
glucose	3+
Ketone	3+
RBC	–

## Investigation

The results of 75-g OGTT at 11 weeks of gestation were 77, 137, and 134 mg/dl at fasting, 1 and 2 h respectively. At the time of admission to our hospital, her arterial pH was 7.45 and base excess was −9.8 mmol/l. Random plasma glucose level was 348 mg/dl with urinary ketones. Her serum sodium (Na) was 132 mmol/l, potassium (K) was 3.8 mmol/l, and chlorine (Cl) was 98 mmol/l, showing that her anion gap was 21.9. Based on these findings, she was diagnosed as having DKA. In addition, diabetes-related antibodies including anti-glutamic acid decarboxylase (GAD) antibody and anti-tyrosine phosphatase (IA-2) antibody were 25.0 and 1.5 U/ml respectively (Table 1). In view of the clinical course and these data, she was diagnosed as having DKA with adult-onset type 1 diabetes mellitus.

## Treatment

**Figure 1:**
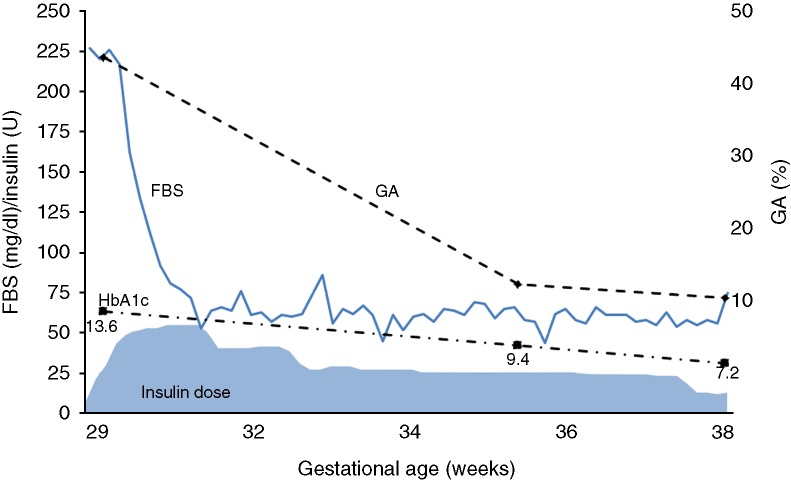
Clinical course. Solid line shows change of fasting blood sugar (FBS) levels, broken line shows change of glycoalbumin (GA) levels, and chain line represents change of HbA1c levels. FBS, fasting blood sugar; GA, glycoalbumin.

After diagnosis of DKA, treatment with saline and intensive insulin therapy was initiated immediately. After therapy, the blood glucose level returned to normal. She had initially needed up to 58 U insulin, but after removing glucotoxicity, only 10 U were needed (Fig. 1).

## Outcome and follow-up

After the ketoacidosis improved, she underwent diet therapy and intensive insulin therapy. She was discharged from the hospital at 34 weeks of gestation. The course of pregnancy up to birth remained uneventful. At 38 weeks of gestation, she spontaneously delivered a healthy baby, which weighed 3.006 g and had an Apgar score of 9/9. Her gestational weight gain was 3 kg. Mother has been receiving insulin therapy at the time of 1.5 years after delivery.

## Discussion

It is unusual for women with a normal GTT in the first trimester to develop type 1 diabetes mellitus later on during pregnancy. In most cases, the carbohydrate profile before or during pregnancy is unknown. However, at 11 weeks of gestation, our patient had normal glucose tolerance. She developed DKA during the third trimester. To the best of our knowledge, this is the first case of a woman with normal glucose tolerance confirmed by 75-g OGTT in the first trimester developing DKA in late pregnancy. Blood tests were positive for anti-GAD antibody and anti-islet cell antibody 2 (ICA2). Therefore, this patient manifested DKA as a result of pregnancy-related autoimmune diabetes. In this case, we focused on differential diagnosis.

Late pregnancy is characterized by a state of insulin resistance. Insulin sensitivity is known to decrease by as much as 56% through 36 weeks of gestation (5). The production of insulin antagonistic hormones such as human placental lactogen, prolactin, and cortisol contributes to insulin resistance. Furthermore, an inflammatory change in adipose tissue is associated with insulin resistance in late pregnancy (6). Therefore, if DKA occurs during late pregnancy, careful diagnosis is required.

There are at least three separate phenotypes of autoimmune diabetes in adults: latent autoimmune diabetes in adults (LADA), adult-onset type 1 diabetes mellitus, and obese patients with phenotypic type 2 diabetes mellitus who are antibody positive (type 1.5) (7). To standardize the definition of LADA, the Immunology of Diabetes Society proposed the following criteria: patients should be at least 30 years of age, positive for at least one of the four antibodies commonly found in type 1 diabetic patients (anti-ICAs, anti-GAD65, anti-IA2, and anti-insulin), and should not have been treated with insulin within the first 6 months of diagnosis.

Fulminant type 1 diabetes mellitus is also a well-known cause of pregnancy-related DKA (8), which is also a characteristic of non-autoimmune diabetes. Fulminant type 1 diabetes mellitus has a rapid onset followed by rapid development of DKA. The level of HbA1c therefore remains nearly normal (9). However, antibodies, including ICAs, anti-GAD65, anti-IA-2, and anti-insulin, are absent in fulminant type 1 diabetes mellitus (9). Our patient had a high level of HbA1c and tested positive for antibodies, thereby ruling out fulminant type 1 diabetes mellitus. After diet and insulin therapy, a target level of glucose could be achieved. In terms of insulin secretion, it is difficult to distinguish between adult type 1 diabetes mellitus and LADA. However, LADA presents clinically slow progression without ketoacidosis and weight loss (10). Therefore, the present case was finally diagnosed as DKA with adult-onset type 1 diabetes mellitus.

In summary, DKA is associated with maternal and fetal mortality. If a patient complaints of general fatigue with urinary glucose and ketones in the latter half of gestation, the presence of DKA caused by the onset of diabetes should be considered, even if the patient showed normal glucose tolerance during the first trimester.

## Patient consent

Written informed consent was obtained from the patient.

## Author contribution statement

All the authors have read the manuscript and have approved this article. H Himuro is the author and T Sugiyama is the corresponding author of this article. H Nishigori, M Saito, J Sugawara, and S Nagase are clinicians who contributed to the management of this case. N Yaegashi is a chief of the department in our hospital.
